# Selective Sparing of Striatal Interneurons after Poly (ADP-Ribose) Polymerase 1 Inhibition in the R6/2 Mouse Model of Huntington’s Disease

**DOI:** 10.3389/fnana.2017.00061

**Published:** 2017-08-02

**Authors:** Emanuela Paldino, Antonella Cardinale, Vincenza D’Angelo, Ilaria Sauve, Carmela Giampà, Francesca R. Fusco

**Affiliations:** ^1^Laboratory of Neuroanatomy, Santa Lucia Foundation IRCCS Hospital Rome, Italy; ^2^Department of Neuroscience, University of Rome Tor Vergata Rome, Italy; ^3^Department of Anatomy and Cell Biology, Catholic University Rome, Italy

**Keywords:** Huntington’s disease, neurodegeneration, PARP-1 inhibition, CBP, pCREB, interneurons, parvalbumin, calretinin

## Abstract

Poly (ADP-ribose) polymerases (PARPs) are enzymes that catalyze ADP-ribose units transfer from NAD to their substrate proteins. It has been observed that PARP-1 is able to increase both post-ischemic and excitotoxic neuronal death. In fact, we have previously shown that, INO-1001, a PARP-1 inhibitor, displays a neuroprotective effect in the R6/2 model of Huntington’s disease (HD). In this study, we investigated the effects of PARP-1-inhibition on modulation of phosphorylated c-AMP response element binding protein (pCREB) and CREB-binding protein (CBP) localization in the different striatal neuronal subsets. Moreover, we studied the neurodegeneration of those interneurons that are particularly vulnerable to HD such as parvalbuminergic and calretininergic, and of other subclasses of interneurons that are known to be resistant, such as cholinergic and somatostatinergic interneurons. Transgenic mice were treated with INO-1001 (10 mg/Kg daily) starting from 4 weeks of age. Double-label immunofluorescence was performed to value the distribution of CBP in ubiquitinated Neuronal intranuclear inclusions (NIIs) in the striatum. INO-1001-treated and saline-treated brain sections were incubated with: goat anti-choline acetyl transferase; goat anti-nitric oxide synthase; mouse anti-parvalbumin and mouse anti-calretinin. Morphometric evaluation and cell counts were performed. Our study showed that the PARP inhibitor has a positive effect in sparing parvalbumin and calretinin-containing interneurons of the striatum, where CREB was upregulated. Moreover, INO-1001 promoted CBP localization into the nuclei of the R6/2 mouse. The sum of our data corroborates the previous observations indicating PARP inhibition as a possible therapeutic tool to fight HD.

## Introduction

Huntington’s disease (HD) is an autosomal dominant neurodegenerative disorder, which is typically characterized by psychiatric disturbances, motor dysfunction and cognitive decline (Wilson et al., 1987). In HD, a gene located on the short arm of chromosome 4 named *IT15* is mutated. IT15 encodes for the protein huntingtin (The Huntington’s Disease Research Collaborative Group, 1993), which mutation leads to a CAG expansion beyond the normal 10–35 triplet repeat range (Albin and Tagle, 1995). From a neuropathological point of view, a dramatic degeneration of neurons in the striatal part of the basal ganglia occurs in HD, accounting for the progressively severe motor dysfunction (Vonsattel et al., 1985; Hedreen et al., 1991; Storey et al., 1992).

Aside from the projection neurons, HD also affects interneurons such as parvalbumin and calretinin-containing neurons. These interneurons appear to degenerate at almost the same rate as the projection neurons (Ferrer et al., 1994). Conversely, the degeneration process relatively spares other subclasses of striatal interneurons, such as somatostatin-NPY and cholinergic interneurons.

Mutated huntingtin impairs the function of cAMP response element-binding protein (CREB; Steffan et al., 2000; Sugars and Rubinsztein, 2003; Sugars et al., 2004), suggesting that inhibition of CREB-mediated gene transcription could play an important role in HD (Kazantsev et al., 1999; Steffan et al., 2000; Nucifora et al., 2001; Mantamadiotis et al., 2002; Jiang et al., 2003). Accordingly, it was observed that cAMP cerebrospinal fluid levels are reduced in HD patients (Cramer et al., 1984) and that CREB-regulated gene transcription is down regulated in the R6/2 HD transgenic mouse (Luthi-Carter et al., 2000; Wyttenbach et al., 2001).

Of note, we previously observed that in parvalbumin-containing neurons, levels of activated CREB are markedly reduced after excitotoxic lesions, which could, at least in part, account for their selective vulnerability (Giampà et al., 2006).

In HD, the N-terminal fragment of mutated huntingtin forms ubiquitinated aggregates named neuronal intranuclear inclusions (NIIs; DiFiglia et al., 1997). These aggregates were shown to be able to interact with several transcription factors, thereby impairing their functions (Martindale et al., 1998; Gutekunst et al., 1999).

CREB binding protein (CBP) is a transcriptional co-activator that was shown to mediate neuronal survival signals (Bonni et al., 1999; Walton and Dragunow, 2000). Moreover, it was observed that NIIs sequestrate CBP in the R6/2 HD mice. Therefore, striatal function is impaired by the interaction between mutated huntingtin and CBP, causing a disruption of transcription, and leading to toxicity for the neuron.

Recently, we showed that a poly (ADP-ribose) polymerase 1 (PARP-1) inhibitor was neuroprotective in the R6/2 mouse model of HD, where sparing of striatal neurons was associated with an increased level of pCREB (Cardinale et al., 2015).

PARP-1, a 116-kD protein, member of PARP family, is a nuclear enzyme, consists of three main domains: the N-terminal DNA-binding domain (DBD), the automodification domain (AMD) and the C-terminal catalytic domain (Kameshita et al., 1984; Kurosaki et al., 1987) involved in the poly(ADP-ribosylation) reaction. The relationship between the inappropriate activation of PARP-1 and neurodegeneration has been demonstrated (Strosznajder et al., 2005; Kauppinen et al., 2011; Martire et al., 2013). In fact, the abnormal activation of PARP induces the release and the translocation of apoptosis-inducing factor (AIF) from the mitochondria to the nucleus causing a programmed cell death, caspase-independent, named parthanatos (Wang et al., 2009).

Many authors have focused the attention on PARP-1 inhibition by common inhibitors used in clinical for cancer treatment, able to restore the physiological cell functions such as mitochondrial activity, or the regulation of transcription factors including p53 required for neuronal survival (Martire et al., 2015).

In an earlier study, our group had shown that, in the R6/2 mouse of HD, the beneficial effects exerted by phosphodiesterase inhibitors on phenotype and on projection neurons sparing was associated to a rescue of parvalbumin positive interneurons and to an inhibition of CBP sequestration into NIIs (Giampà et al., 2009).

The aim of the present study was to deepen our knowledge of the effects of PARP-1-inhibition. In particular, we aimed at investigating the effects of PARP-1 inhibition on CBP localization, by reducing CBP sequestration into the NIIs, and thus decreasing cellular toxicity resulting from it. Moreover, we studied the effects of the compound on calretinin and parvalbumin containing striatal cells, two subsets of interneurons that are particularly vulnerable to HD. In summary, our aim was to verify if the modulation of CBP activity (Oliveira et al., 2006), on one hand, and the sparing of selected striatal interneurons (Torres et al., 1994), on the other, were associated with the previously described neuroprotective effects and the rescue motor deficits in HD mice.

## Materials and Methods

### Animals and PARP-1 Inhibitor Administration

All studies were performed in accordance with European Communities Council Directive of 24 November 1986(86/609/EEC) as adopted by Santa Lucia Foundation Animal care and Use committee. The Santa Lucia Foundation Animal Care and Use committee approved this study.

The animal model of HD, R6/2, was employed for this study. Transgenic R6/2 mice were obtained by ovarian transplantation of hemizygote females X B6CBAF1/J males, provided by Jackson laboratories (Bar Harbor, ME, USA) and the F1 mice were used to perform all the experiments. Animals were genotyped by PCR assay of DNA obtained from tail tissue. After genotyping at 4 weeks of age, mice were weaned and the treatment started. The treatment was continued until sacrifice. The criterion for euthanasia was that the mouse was not capable to right itself when placed on a side (after 13^th^ week of age). Twenty-four mice per group were used. The study groups were composed by: R6/2 and wild type mice treated with 0.9% saline administered by intraperitoneal injection and R6/2 and wild type mice with INO-1001 dissolved in saline (10 mg/Kg/day). INO-1001 was diluted immediately before use and administered twice in the same day. Mice were housed five in each cage under standard conditions with *ad libitum* access to food and water.

### Tissue Processing

Experimental animals were transcardially perfused under deep anesthesia with Zoletil and Rampun (500–800 mg/Kg), followed by 60 ml of 4% paraformaldehyde in saline solution. Brains were removed and post fixed overnight at 4°C, cryoprotected in 10% sucrose and 20% glycerol 0.1 M phosphate buffer (PB) with 0.02% sodium azide for 48 h at 4°C. Subsequently, all brains were sectioned on sliding microtome at 40 μm thickness to obtain serial sections.

### Immunohistochemical Studies

#### Analysis of NIIs in Neurons Expressing CBP

Double-label immunofluorescences were carried out to evaluate the distribution of ubiquitinated NIIs according to CREB binding protein (CBP) expression levels in the striatum. Coronal brain sections of mice treated with PARP-1 inhibitor and vehicle were incubated with a cocktail of anti-CBP antibody (rabbit anti-CBP, Immunological Sciences, RM, Italy) and a mouse antibody against mutant huntingtin protein (Clone mEM48, Merk Millipore Corporation, Darmstadt, Germany) at 1:200 dilution in a 0.1 M PB solution containing 0.3% Triton X-100 for 72 h at 4°C, except for CBP protein which needs the antigen retrieval method. Antigen retrieval was performed in Citrate Buffer (pH 6) for 20 min at 80°C. After that, sections were retained in this buffer solution while allowing it to cool at room temperature. Sections were rinsed three times for 5 min at room temperature and subsequently incubated with the primary antibody against CBP. The immunohistochemical staining was completed with the streptavidin-biotin amplification and the goat anti-mouse secondary antibody for EM48 (Jackson Immunoresearch, West Grove, PA, USA) for 2 h at room temperature at 1:100 dilution in a 0.1 M PB solution containing 0.3% Triton X-100. Sections were mounted on gelatin-coated slides, cover slipped with GEL-MOUNT (Sigma-Aldrich, Italy) and a confocal laser scanning microscope (Zeiss LSM 510) was used to acquire all of the images. Three separate fields (dorsolateral, central and medial each 1 mm in diameter) on each hemisphere in each of three rostro caudally spaced sections in each of four mice per group were examined. NIIs have been quantified and measured by using the Java image processing and analysis program; ImageJ. Cells of interest were selected using the freehand tool. From the Analyze menu, measurements were set “Mean Grey Value, Area and Min and Max Grey Value” were selected. Finally, the mean values of all measures were obtained. Analysis of NIIs size and number was performed by the operator in blind to treatment.

#### Striatal Interneurons Characterization

A single immunological staining for striatal interneurons markers, counterstained with Neurotrace fluorescent Nissl (green fluorescence) and DAPI was performed. INO-1001 and saline-treated brain sections were incubated with goat anti-choline acetyl transferase (ChAT; Nova biological, CA, USA); goat anti-nitric oxide synthase (NOS; Sigma, St. Louis, MO, USA); mouse anti-parvalbuminergic (PARV, Chemicon International, Inc., Temecula, CA, USA) and mouse anti-calretinin (CALR; Chemicon International, Inc., Temecula, CA, USA).

All primary antibodies were used at a 1:200 dilution, in 0.1 M PB containing 0.3% Triton X-100 for 72 h at 4°C. Section were rinsed three times for 5 min at room temperature and subsequently incubated with secondary antibodies: donkey anti-mouse and donkey anti-goat (Jackson Immunoresearch, West Grove, PA, USA) for 2 h at room temperature at 1:100 dilution in a 0.1 M PB solution containing 0.3% Triton X-100. Subsequently sections were mounted on slides, cover slipped with GEL-MOUNT and examined under an epi-illumination fluorescence microscope (Zeiss Axioskop 2). The confocal laser scanner microscopy (Zeiss LSM700) was used to acquire images.

#### Study of Parvalbuminergic Neurons

Peroxidase-antiperoxidase diaminobenzidine tetrahydrochloride single-label immunohistochemistry for parvalbumin (PARV) was performed to identify and count parvalbuminergic interneurons in the striatum. Serial sections from rostral neostriatum through the level of anterior commissure (interaural 4.66 mm/Bregma 0.86 mm to interaural 3.34 mm/Bregma −0, 0.46 mm) for three animals per groups, were incubated with mouse anti-PARV at 1:200 dilution in 0.1 M PB solution containing 0.3% Triton X-100 for 72 h at 4°C. Subsequently sections were incubated with mouse peroxidase-antiperoxidase complex diluted 1:100 in 0.1 M PB solution with 0.3% Triton X-100 at room temperature for 1 h. After peroxidase-antiperoxidase incubation, sections were incubated in Tris-Hcl buffer containing 10 mg diaminobenzidine tetrahydrochloride for 5 min, adding 15 μl of 3% hydrogen peroxidase. The peroxidase-antiperoxidase diaminobenzidine tetrahydrochloride-labeled sections were then washed in distillated water, placed in 0.1 M PB, mounted on gelatin-coated slides, dried, dehydrated and coverslipped. PARV positive cells count was performed using Neurolucida^TM^ Stereo Investigator software (Zeiss, Rochester, NY, USA).

#### pCREB Expression and Quantification

The dual-label immunofluorescence was employed to assess the expression pattern of phosphorylated CREB in the striatal interneurons population.

Sections were incubated with a cocktail of rabbit anti-pCREB (Millipore Corporation, RM, Italy) and one of the striatal interneuron markers: ChAT, NOS, CALR and PARV. Tissue was mounted on slides, coverslipped with GEL-MOUNT and the confocal laser scanner microscopy (Zeiss LSM700). For these experiments, parameters set on sections of control mice (Wt), such as laser %, pinhole and gain master, were equal during images acquisition.

### Statistical Analysis

#### Single and Double Immunolabeling

All the collected images have been quantified by using the Java image processing and analysis program ImageJ. Cells of interest were selected using the freehand tool. From the Analyze menu, Set measurements Mean “Grey Value”, “Area” and “Min and Max Grey Value” were selected. The region characterized by absence of fluorescence was considered in the background and it was subtracted. Finally, the mean values with SEM were obtained for all measures. ANOVA analysis available in the software Stat version 12 and GraphPad Prism version 7.0 was performed. *P* values of less than 0.05 were considered statistically significant.

## Results

### INO-1001 Prevents the Sequestration of CBP

**Figure 1:**
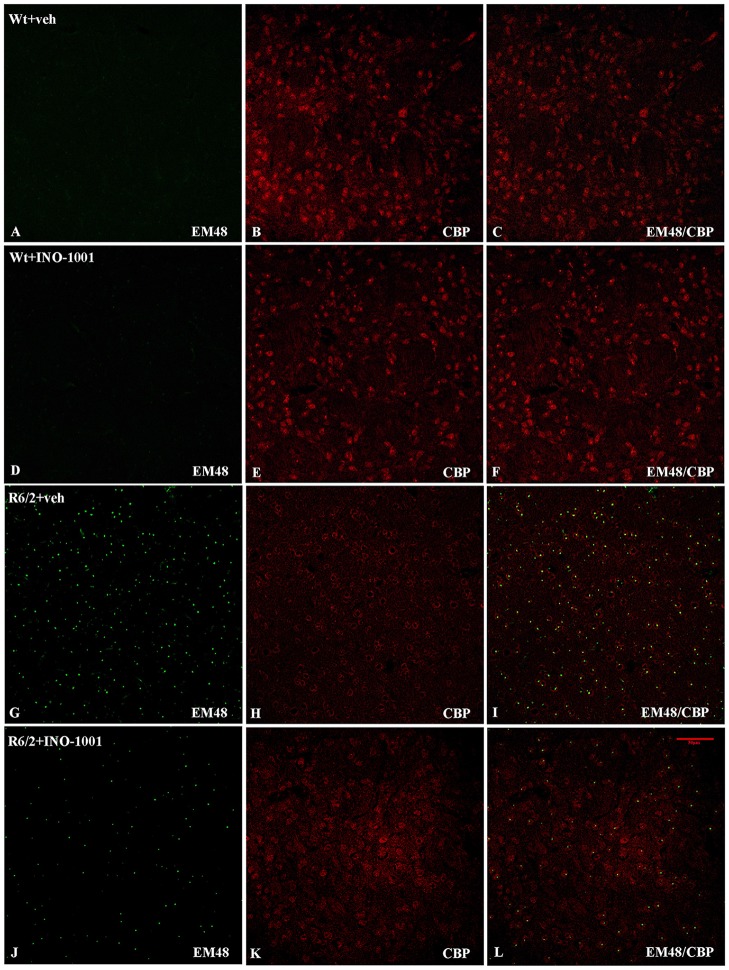
Double-label immunofluorescence for CREB-binding protein (CBP) and Neuronal intranuclear inclusions (NIIs). The double label immunostaining was performed in Wt mice **(A–C)**, INO-1001 treated Wt **(D–F)**, vehicle treated R6/2 mice **(G–I)** and INO-1001 treated mice **(J–L)**. CBP is labeled in red and NIIs in green. NIIs are not found in the WT groups **(A,D)**. In **(J)** please observe the low density, reduced size and immunolabeling intensity of NIIs in R6/2 treated with INO- 1001, and the CBP expression pattern similar to Wt groups.

CBP localization and expression in R6/2 transgenic mice treated with saline or INO-1001 were investigated. A specific antibody against mutated huntingtin revealed NIIs in R6/2 mice and the CBP immunoreactivity in each group was analyzed. Figures 1A–F show the physiological (mostly nuclear) distribution of CBP in wild type mice, which exhibit normal morphological features and vitality. A strong depletion of CBP was observed in the nuclei of saline-treated R6/2 mice (Figures 1G–I).

### NIIs Are Reduced after PARP Inhibition

**Figure 2:**
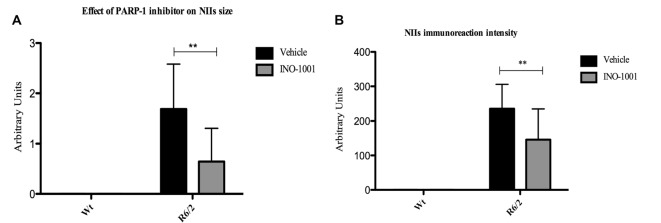
The effect of Poly (ADP-ribose) polymerase-1 (PARP-1) inhibitor on NIIs size and intensity. Two-way ANOVA performed on data obtained by vehicle and INO-1001 treated R6/2 mice revealed a statistically significant effect of treatment on NIIs size and intensity. **(A)** *Bonferroni* analysis showed a significant decrease of NIIs size *p* < 0.001 (*F*_(1,28)_ = 7.019; *P* = 0.0031) and intensity in mice treated with PARP-1 inhibitor respect to vehicle treated R6/2 *p* < 0.001 (*F*_(1,28)_ = 4.96; *P* = 0.0060; Panel **B**).

As expected, wild type mice striata did not show any immunoreactivity for NIIs. On the contrary, striatal brains of 11-week-old R6/2 mice showed, as previously demonstrated, an elevated number of NIIs [31] (Figures 1G–I). As mentioned above, the NIIs positive nuclei did not show CBP expression in saline-treated R6/2 mice compared to INO-1001-treated mice (Figures 1J–L). Moreover, the analysis of all saline-treated R6/2 revealed that the NIIs area and immunoreaction intensity were decreased compared to cells of INO-1001 treated mice (Figures 2A,B). Daily administration of PARP-1 inhibitor showed a reduced NIIs density, with a significant decrease in NIIs size. Treatment promoted the CBP expression restoration, showing a CBP staining pattern in R6/2 mice brains sections that was comparable to that of wild type animals.

### INO-1001 Prevents the Loss of Parvalbumin- and Calretinin Containing Interneurons

**Figure 3:**
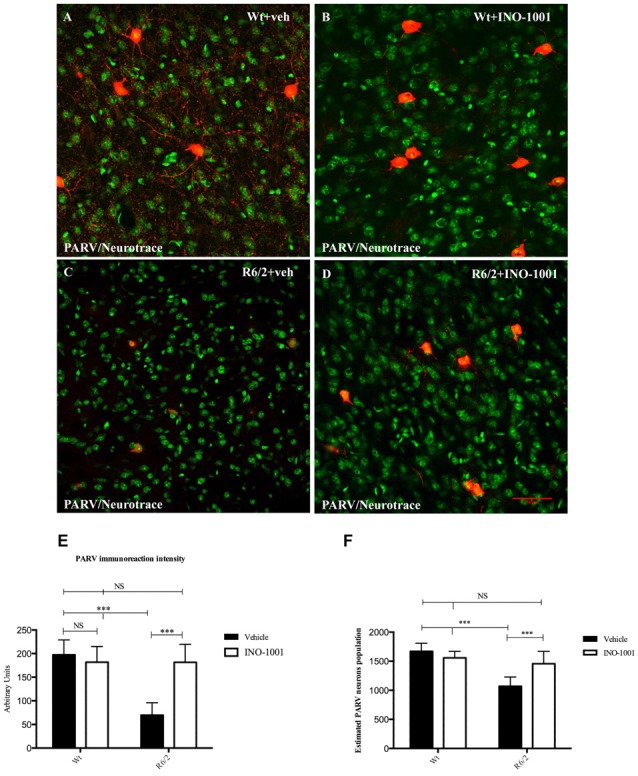
Confocal laser scanning microscopy images of double-label immunofluorescence for PV (visualized in red-Cy3 fluorescence) and the Nissl-like fluorescent marker Neurotrace (visualized in green fluorescence) **(A–D)**. Graphs show **(E)** the marked effect of INO-1001 administration in R6/2 mice striatal PARV neurons. Analyzed data revealed the significant effect of PARP-1 inhibitor (*p* < 0.0001) in promoting the parvalbumin-containing neurons protection. INO-1001 treatment had no effect in Wt mice. Densities of all interneuron subtypes markers were compared by means of two-way ANOVA with *Bonferroni* analysis (*F*_(1,28)_ = 6.041; *p* = 0.0004). **(E–F)** Single-label diaminobenzidine tetrahydrochloride immunohistochemistry for PV in the dorsolateral striata of vehicle-treated **(E)** and INO-1001-treated **(F)** R6/2 mice. Graph **(F)** shows the statistically significant effect of INO-1001 daily administration on the most vulnerable striatal interneurons subtype in R6/2 mice.

Striatal PARV interneurons of wild-type animals administered either saline or INO-1001 were medium sized with axons with very dense arborizations (Figure 3). The density of PARV interneurons was markedly reduced in the vehicle-treated R6/2 mice, with a smaller cell body and fewer or no arborizations. INO-1001 increased the density of PARV interneurons in the R6/2 mice compared to saline treatment (Figure 3). The Anova test revealed a significant effect of the treatment. Bonferroni analysis showed no effect on the number of PARV interneurons in the wild-type mice treated with INO-1001 and a protective effect on the striatal parvalbumin-containing neurons of R6/2 mice.

**Figure 4:**
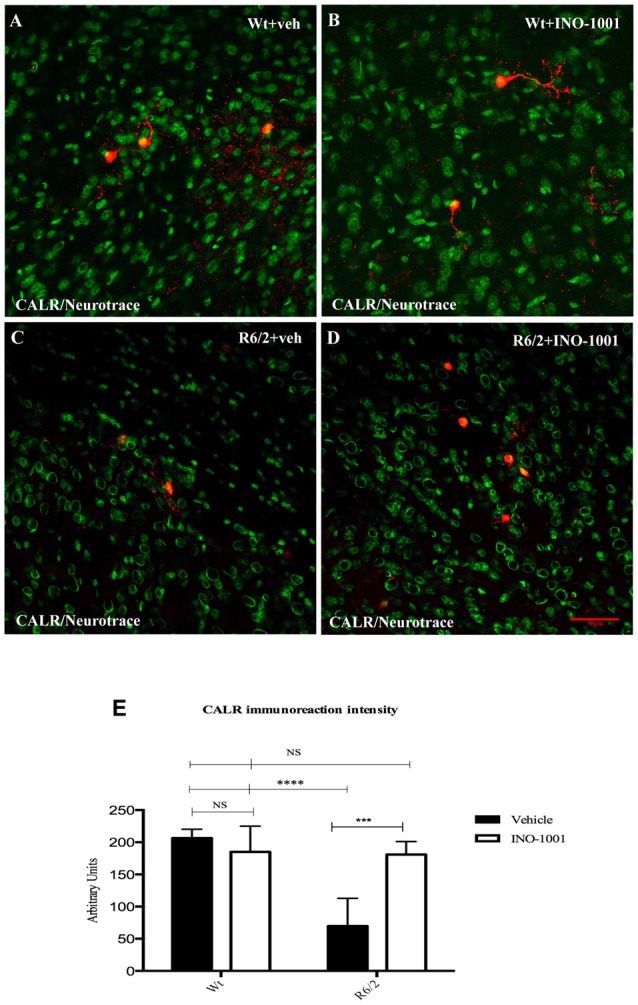
Confocal laser scanning microscopy images of double-label immunofluorescence for CALR (visualized in red-Cy3 fluorescence) and the Nissl-like fluorescent marker Neurotrace (visualized in green fluorescence) **(A–D)**. The graph **(E)** shows the marked effect of INO-1001 administration in R6/2 mice striatal CALR neurons. Analyzed data revealed the significant effect of PARP-1 inhibitor in promoting the calretinin-containing neurons protection. INO-1001 treatment had no effect in Wt mice. Densities of all interneuron subtypes markers were compared by means of two-way ANOVA with *Bonferroni* analysis (*F*_(1,28)_ = 12.56; *P* = 0.0001).

Striatal interneurons were identified by the specific immunoreactivity markers ChAT, NOS, CALR and PARV. Collected data confirmed the previous observations about the survival of specific striatal interneurons subtypes in R6/2 mice and a specific vulnerability of CALR and PARV positive interneurons in the late states of HD. The single immunolabeling study showed an equal distribution of ChAT and NOS interneurons in all experimental groups (data not shown), compared to PARV and CALR positive neurons, which exhibited a dramatically reduced number, and immunoreactivity intensity, in saline- treated R6/2 mice. However, INO-1001 treatment promoted a significant increase in number of both PARV and CALR interneurons and arborizations (Figure 4). The density of PARV and CALR interneurons was comparable to that of wild type mice.

### Analysis of pCREB Expression Levels in the Interneurons Subtypes

**Figure 5:**
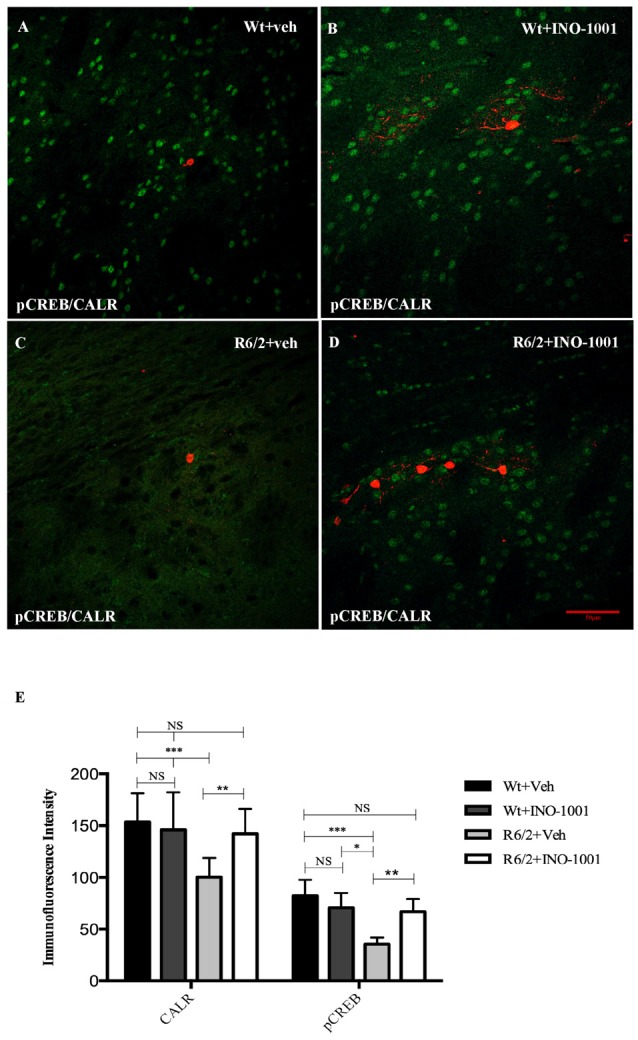
Confocal images of double-label immunofluorescence for phosphorylated c-AMP response element binding protein (pCREB) and Calretinin. pCREB is showed in green CY-2 fluorescence, Calretinin is labeled in red Cy-3 **(A–D)**. Graph **(E)** shows the immunoreaction intensity relative to pCREB and Calretinin in each animal groups. A two-way ANOVA with genotype and treatment as main factors revealed that R6/2 mice had a significant impairment in pCREB expression levels in the Calretinin positive interneurons R6/2 compared to vehicle and INO-1001-treated wild-type (*F*_(1,28)_ = 29.625; *P* = 0.0001). INO-1001 treatment restored pCREB expression in Calretinin-containing neurons in a genotype dependent fashion *F*_(1,28)_ = 21.523; *P* = 0.0001.

**Figure 6:**
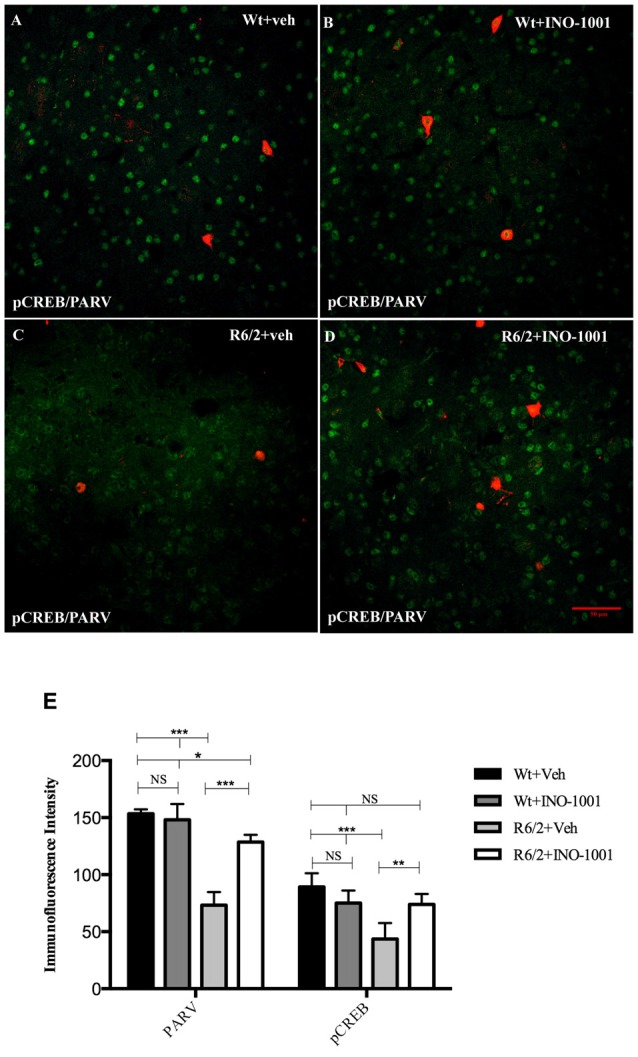
Confocal images of double-label immunofluorescence for pCREB and Parvalbumin. pCREB is labeled in green CY-2 fluorescence, Parvalbumin in red Cy-3 **(A–D)**. Graph shows the immunoreaction intensity relative to pCREB and Parvalbumin in the different experimental animal groups **(E)**. pCREB content is lower in the vulnerable parvalbuminergic interneurons of saline treated R6/2 mice respect to R6/2 treated with INO-1001 (*F*_(1,28)_ = 67.837; *P* = 0.0000) that increases pCREB and parvalbumin expression levels in a genotype dependent fashion (significant genotype × treatment interaction pCREB *F*_(1,28)_ = 32.889; *P* = 0.0010; PARV *F*_(1,28)_ = 57.123; *P* = 0.0000).

PARP-1 inhibitor, INO-1001, promoted a rescue in pCREB expression in R6/2 mice compared to saline-treated mice [25]. The intensity of phosphorylated CREB immunoreaction product in the interneurons subtypes was investigated in all experimental groups. The present study shows that pCREB expression was decreased in CALR interneurons of saline treated R6/2 mice, where it was up regulated by INO-1001 treatment (Figure 5). A comparable result was obtained in PARV neurons, where INO-1001 significantly increased pCREB content in the R6/2 mice (Figure 6).

## Discussion

Our findings demonstrate that chronic treatment with the PARP inhibitor INO-1001 has a protective effect for striatal parvalbumin and calretinin-containing neurons in terms of neurodegeneration, and prevents CBP sequestration into NIIs.

Neurodegeneration affects neuronal subpopulations of the striatum in different ways. In fact, PARV-positive GABAergic interneurons display a cell vulnerability that is comparable to that of projection neurons (Ferrer et al., 1994; Liang et al., 2005). Notably, projection neurons and interneurons are different in their sensitivity to both ischemic and excitotoxic insults (Roberts and DiFiglia, 1989; Chesselet et al., 1990). However, the molecular basis of such different behavior remains to be fully elucidated. (Beal et al., 1989; Figueredo-Cardenas et al., 1994). One possibility that our group had investigated was that some interneurons tendency to survive longer (such as cholinergic interneurons) was related to their ability to maintain sufficient levels of BDNF (Fusco et al., 2003).

Striatal parvalbumin interneurons are GABAergic neurons that have a spiny varicose dendrites and indented nuclei (Celio, 1990; Bennett and Bolam, 1994) and axons with typically dense collateral arborizations (Cicchetti and Parent, 1996) and because they fire phasically at high frequency in response to cortical stimulation, they are considered fast-firing neurons (Kita, 1993; Kawaguchi et al., 1995). Striatal PV neurons are considered interneurons because of their morphological characteristics, which include a medium-sized cell body, aspiny varicose dendrites and a strongly indented nucleus (Celio, 1990; Bennett and Bolam, 1994).

GABAergic PV-positive interneurons only constitute 3%–5% of the cells in the rodent neostriatum (Kawaguchi et al., 1995), and yet they are able to exert a very powerful inhibitory control on the activity of cortical projecting neurons (Koos and Tepper, 1999). Cepeda et al. (2013) described how such a small population of neurons plays a pivotal role in HD. Indeed, by means of optogenetics and electrophysiology, they described that parvalbumin neurons are an important source for increased frequency of spontaneous GABA synaptic activity in the striatum.

Interestingly, these neurons are not only as vulnerable as projection neurons to HD but they also tend to form NIIs to the same extent as projection neurons, whereas other types of more resistant interneurons, such as cholinergic and somatostatin interneurons, form NIIs less frequently (Kosinski et al., 1999). Thus, the observation that PARP inhibition significantly spared the striatal PARV interneurons confirms the beneficial effect of the treatment on HD pathology.

However, a sparing of parvalbumin neurons could be desirable not only for HD, but also in other hyperkinetic neurological disorders. Indeed, striatal parvalbumin expression is decreased in a dystonic hamster model (Bode et al., 2017). Thus, disorders where parvalbumin interneurons fast spiking activity is involved such as dystonia could benefit from such neuroprotective effect (Reiner et al., 2003).

Calretinin is a calcium-binding protein containing a structural domain that binds calcium (Henzi et al., 2009). A protective role for calretinin has been postulated, as it was observed that CALR neurons are spared in a 6-OHDA-lesion model (Tsuboi et al., 2000) and also in HD patients (Massouh et al., 2008). Moreover, CALR was shown to be correlated to an impaired neurogenesis in adult HD (Fedele et al., 2011). In a recent report, Dong et al. (2012) demonstrated that CALR can interact with mutant huntingtin and that it can decrease cytotoxicity caused by mutant huntingtin in cellular models of HD.

We observed that CALR neurons were rescued by INO-1001 treatment, supporting the idea that functionally active CALR interneurons may participate in the survival of striatum in HD.

The observation that PARP-inhibition was effective in decreasing the size of NIIs was in agreement with our previous report about the reduced frequency of NIIs in the INO-1001 treated mice. The role of NIIs in HD has been discussed for many years now, and the question whether aggregates could be the basis of clinical manifestations and neurodegeneration in HD still remains unsolved (Davies and Scherzinger, 1997; Kim and Tanzi, 1998; Saudou et al., 1998; Sisodia, 1998). It is conceivable that HD aggregates could trigger cellular dysfunction leading to the death of those neurons in which they are contained. Indeed, NIIs formation can impair cellular functions by their ability to sequester proteins and transcription factors, thereby causing a dysfunction of a number of cellular transcriptional mechanisms (Maat-Schieman et al., 1999; Perutz, 1999; Preisinger et al., 1999; Meade et al., 2000). However, there is no clear demonstration that aggregates are the direct cause of HD neuronal death. Moreover, Saudou et al. demonstrated that neuronal death caused by the mutation is not directly related to NIIs formation in cultured cells transfected with mutant huntingtin. Additionally, Reiner and co-workers (Reiner et al., 1988) observed that the presence of NIIs was related to a prolonged survival in chimeric R6/2 mice.

However, our studies have shown that every treatment that proved beneficial for HD phenotype and neurodegeneration (DeMarch et al., 2008) was also effective in decreasing the frequency and the size of NIIs. Moreover, a toxic effect for aggregates was demonstrated. Therefore, polyQ aggregation could be considered as a therapeutic target for HD (Bates, 2003).

Transcriptional responses to cAMP are mediated by the interaction of CREB with CBP, and cAMP activated transcription is regulated by CREB phosphorylation (Azuma et al., 1996).

However, CBP is not only a transcriptional coactivator for CREB (Arany et al., 1994). Indeed, CBP is able to acetylate promoter proximal nucleosome histones, which results in an augmented availability of DNA for other important factors (Ogryzko et al., 1996). Of note, several studies clearly demonstrated that histone acetyltransferase activity could be altered by mutated huntingtin. Moreover, the enzyme dysfunction could be at the basis of the transcription deregulation occurring in HD. Interestingly, histone deacetylase inhibitors have proven to display a beneficial effect in several HD models (Sadri-Vakili and Cha, 2006).

CBP is abundant in the nuclei of 100% of wild type cells, but only in 18% of HD mutant cells (Gines et al., 2003). The nuclear localization of CBP is therefore a feature of normal cells, and its displacement away from the nucleus and sequestration into the aggregates accounts for the cellular dysfunction in HD. The phenomenon described is indeed typical of HD pathology, as it has been observed in several HD models, from cell cultures to human patients (Nucifora et al., 2001). An association between the depletion of CBP and mutant huntingtin-mediated cellular toxicity was previously demonstrated (Jiang et al., 2006).

Therefore, the physiological role of CBP is markedly impaired in HD and it participates in the neuronal degeneration not only via the altered CREB function but also through the detrimental effect on the mechanism of histone acetylation. Correspondingly, the improved functioning of CBP might be related not only to the restored CREB functions, but also to rescued physiological functioning of CBP *per se*.

Accordingly, we are reporting that INO-1001 is able to prevent CBP segregation into the aggregates. This confirms our recent report on neuroprotection exerted by INO-1001 in the R6/2 mice, and supporting the idea that reinstating the nuclear localization of CBP is able to participate to the correct cellular functioning. In line with our earlier report, we can establish that there is a strong correlation between CBP recruitment in NIIs and neuronal death.

Thus, we can speculate that the reinstated CREB function, along with the physiological localization of CBP in the nucleus, can concur in keeping adequate BNDF expression levels that are necessary to protect striatal neurons from HD neurodegeneration (Bemelmans et al., 1999; Pérez-Navarro et al., 2000).

We also provide the first evidence that parvalbumin and calretinin-containing interneuron are rescued by PARP inhibition in the R6/2 mouse model of HD. Therefore, these results demonstrate the possibility of ameliorating HD neuropathology by chronic peripheral administration of INO-1001 and, more generally, underline the potential therapeutic value of PARP inhibitors in HD.

## Author Contributions

FRF designed the experimental plan and wrote the article. CG performed experiments and statistical analysis. EP performed experiments and wrote the the results. IS performed experiments. AC performed experiments. VDA performed experiments.

## Conflict of Interest Statement

The authors declare that the research was conducted in the absence of any commercial or financial relationships that could be construed as a potential conflict of interest. The reviewer NM declared a shared affiliation, though no other collaboration, with one of the authors VD to the handling Editor, who ensured that the process nevertheless met the standards of a fair and objective review.
